# Falling apart

**DOI:** 10.7554/eLife.18203

**Published:** 2016-06-27

**Authors:** Jonathan S Marvin, Loren L Looger

**Affiliations:** Janelia Research Campus, Howard Hughes Medical Institute, Ashburn, United Statesmarvinj@janelia.hhmi.org; Janelia Research Campus, Howard Hughes Medical Institute, Ashburn, United Statesloogerl@janelia.hhmi.org

**Keywords:** nanobodies, GFP, HIV-1, antibody engineering, Human, Mouse

## Abstract

Destabilized nanobodies can be used to deliver fluorescent proteins and enzymes to specific targets inside cells.

**Related research article** Jonathan CY Tang, Eugene Drokhlyansky, Behzad Etemad, Stephanie Rudolph, Binggege Guo, Sui Wang, Emily G Ellis, Jonathan Z Li, Constance L Cepko. 2016. Detection and manipulation of live antigen-expressing cells using conditionally stable nanobodies. *eLife* **5**:e15312. doi: 10.7554/eLife.15312

**Image** Nanobodies highlight cells of interest in the mouse retina

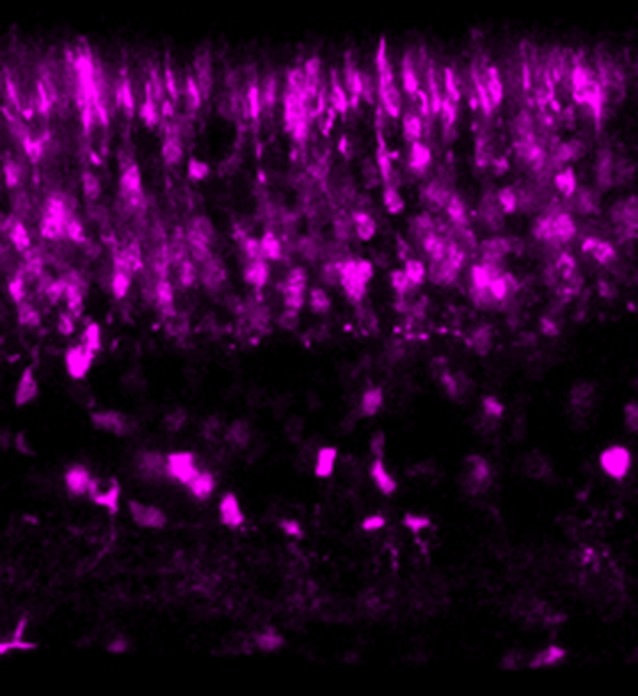

Antibodies are generally quite stable molecules. This is almost always a good thing for their "day job", which is to circulate in the blood and fight off infections. The ability of antibodies to target specific proteins or populations of cells can also be exploited in a range of cell biology experiments. Over the last two decades, researchers have developed various biomolecules that can be expressed inside cells to take the place of antibodies in these experiments. The biomolecules are designed to have the targeting properties of antibodies without suffering some of the drawbacks associated with them.

Biomolecules called nanobodies have emerged as one of the best replacements for antibodies. (Nanobodies are naturally produced as part of the adaptive immune systems of camelids, including camels and llamas, and cartilaginous fish, such as sharks and rays.) A nanobody can, for example, be combined with green fluorescent protein (GFP) to make a “fusion protein” that can target a particular protein in imaging applications.

However, gathering meaningful data from nanobody-based fusion proteins is not as easy as it seems. One issue is that the stability of nanobodies within cells means that many more fusion proteins are expressed than are needed to bind to the target. This is a problem in imaging applications, for example, because fusion proteins that do not bind the target will still produce fluorescence. What is required is a feedback mechanism for the cell to produce only as many nanobodies as are needed to decorate the target. Now, in eLife, Connie Cepko and colleagues at Harvard Medical School – including Jonathan Tang and Eugene Drokhlyansky as joint first authors – describe such a system (Tang et al., 2016).

The key insight of the work was to create nanobody mutants that fold well and are stable when bound to their target, but become floppy and unstable when not bound to their target (Figure 1). This allows the cell to degrade the unstable nanobodies (along with any proteins to which they are fused). This is a tricky proposition: if the nanobody is too stable, it will persist when unbound, but if it is compromised too much, it may be degraded immediately after it has been produced.

**Figure 1.**
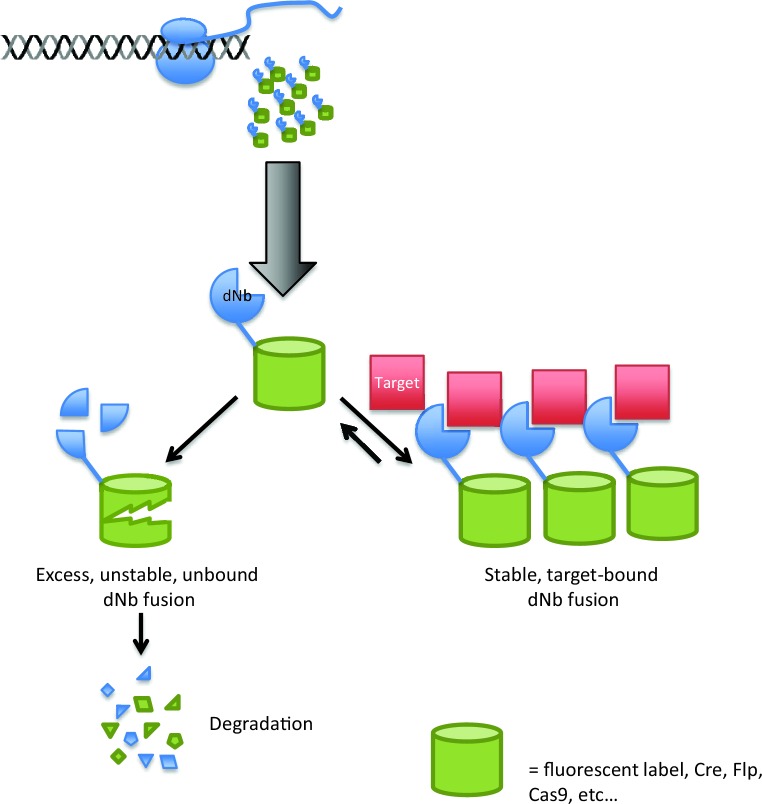
Using destabilized nanobodies for the targeted labeling or manipulation of proteins within live cells. The ability of an antibody to target a specific molecule can be exploited in a range of cell biology experiments, but expressing antibodies in living cells (top) can be difficult. Using a destabilized nanobody (dNb; blue) can overcome some of these problems (Tang et al., 2016). Destabilized nanobodies can be used to deliver fluorescent labels or specific enzymes (green cylinders) to specific targets (red; right). If the target is already saturated by nanobody, the rest of the nanobody is degraded (left), thus preventing unwanted background fluorescence or enzyme activity.

Building on previous work on “conditionally stable” proteins – proteins that have been designed so that they are only stable in the presence of a small molecule (Banaszynski et al., 2006) – Tang et al. began by investigating a nanobody called GBP1, which is one of the best characterized of all nanobodies (Kirchhofer et al., 2010). Through a handful of clever screens, they isolated a form of GBP1 called destabilized GBP1 that binds to GFP (bringing along any fused proteins as well), but is quickly degraded in its absence.

Importantly, the mutations that destabilized GBP1 occurred away from the region that binds to GFP. Given that the sequence of this region is highly similar across different nanobodies, Tang et al. reasoned that the destabilizing mutations might be transferable to other nanobodies. This indeed was the case, and they were able to make conditionally stable nanobodies for seven different target proteins.

Tang et al. first fused fluorescent proteins to the destabilized nanobodies, which allowed various target proteins to be visualized inside cultured cells, retinas and brain slices. With some more tweaking, the destabilized nanobodies worked equally well when fused to the CRISPR-Cas9 gene editing enzyme (Doudna and Charpentier, 2014) and to various DNA-modifying enzymes (such as the Flp and Cre recombinases; Birling et al., 2009). The resulting fusion proteins were able to access cells that contained target proteins with a particular amino acid sequence. Since these sequences are much more highly conserved across organisms than their underlying nucleic acid sequences, destabilized nanobodies could be used to make genetic modifications to a wide variety of organisms, including organisms that are poorly served by existing genetic toolboxes.

Tang et al. also demonstrated that their fusion proteins can be used as diagnostic tools. They were able to label HIV-infected cells without the need for laborious identification methods, such as immunolabeling, sequencing or polymerase chain reaction (PCR)-based techniques. This means that reagents based on destabilized nanobodies could help to diagnose a variety of pathogens and metabolic states at the point of care.

Much work remains to be done. Just a few active Cre or Cas9 molecules can act on all the genomic targets within a cell, so the background activity of these molecules must be tightly clamped at zero. To date no system has achieved this level of regulation, but an approach based on destabilized nanobodies certainly seems to be an improvement over approaches in which drugs are used to control the activity of Cre enzymes (Zhang et al., 2012).

There are other approaches for regulating the expression of antibodies (and related binding domains) within cells. For example, adding a transcriptional repressor domain to certain fusion proteins creates a negative feedback loop, whereby excess fusion protein represses its own production (Gross et al., 2013). This system has recently been extended to fusion proteins that include E3 ubiquitin ligases and used to degrade target proteins (Gross et al., 2016).

The complement of well-validated nanobodies and related binding domains continues to grow, and structural information allows researchers to see how target recognition occurs at the atomic level. Sequencing projects are producing large repositories of target proteins in a host of settings, including neuroscience model organisms, human cell types and the many pathogens that threaten public health. All in all, the future is very promising for the conditional manipulation of targeted proteins and cells within complex organisms.
